# Technical success and initial clinical outcome of partial frozen elephant trunk in various aortic arch pathology

**DOI:** 10.1007/s12055-025-02054-y

**Published:** 2025-09-15

**Authors:** Takeshi Shimamoto, Kenji Minatoya, Tatsuhiko Komiya, Nobuhisa Ohno, Nobushige Tamura, Hitoshi Sakaguchi, Kyokun Uehara, Hiroshi Tsuneyoshi, Jiro Esaki

**Affiliations:** 1 Department of Cardiovascular Surgery, Hamamatsu Rosai Hospital, 25 Shogen-Cho, Chuo-Ku, Hamamatsu, Shizuoka, 430-8525 Japan; 2 Department of Cardiovascular Surgery, Kyoto University Hospital, Kyoto, Japan; 3 Department of Cardiovascular Surgery, Kurashiki Central Hospital, Kurashiki, Okayama, Japan; 4 Department of Cardiovascular Surgery, Kokura Memorial Hospital, Kitakyushu, Fukuoka, Japan; 5 Department of Cardiovascular Surgery, Hyogo Prefectural Amagasaki General Medical Center, Amagasaki, Hyogo Japan; 6 Department of Cardiovascular Surgery, Japan Red Cross Society Wakayama Medical Center, Wakayama, Japan; 7 Department of Cardiovascular Surgery, Tenri Hospital, Tenri, Japan; 8 Department of Cardiovascular Surgery, Shizuoka General Hospital, Shizuoka, Japan; 9 Department of Cardiovascular Surgery, Kobe Central Municipal Hospital, Kobe, Japan

**Keywords:** Frozen elephant trunk, Aortic dissection, Pseudoaneurysm, Surgery

## Abstract

**Objective:**

This study aimed to investigate the operative and short-term clinical outcomes of total arch replacement using the partial frozen elephant trunk (FET) technique.

**Methods:**

Sixteen patients with aortic arch disease were treated with partial FET at six institutions between August and December 2023. The primary endpoint of our study was the technical success of partial FET implantation. The secondary endpoints included death and the occurrence of aortic events, such as the distal stent graft–induced new entry. Numerical values are presented as mean ± standard deviation.

**Results:**

The mean age of the patients was 62 ± 10 years, with eight being female. The indications for the operation included acute aortic dissection in 10 patients, chronic aortic dissection in 5, and anastomotic pseudoaneurysm in 1. Total arch replacement with partial FET was successfully performed on all patients. The duration of circulatory arrest, aortic cross-clamping, and cardiopulmonary bypass were 62 ± 17, 176 ± 66, and 271 ± 65 min, respectively. No symptomatic postoperative pressure gradient between the upper and lower extremities was observed. The 30-day and in-hospital mortality rates were both 0%. There were no cases of new postoperative stroke, paraplegia, or paraparesis. Additionally, no aorta-related complications were reported. An excellent surgical view of the distal unstented portion of the partial FET was achieved through left thoracotomy during the thoracoabdominal aortic replacement performed 3 months after the initial surgery.

**Conclusions:**

The clinical outcomes of total arch replacement using the partial FET technique were satisfactory. However, surgeons must exercise caution when employing this method for chronic aortic dissection due to its weak radial and spring back forces.

**Graphical Abstract:**

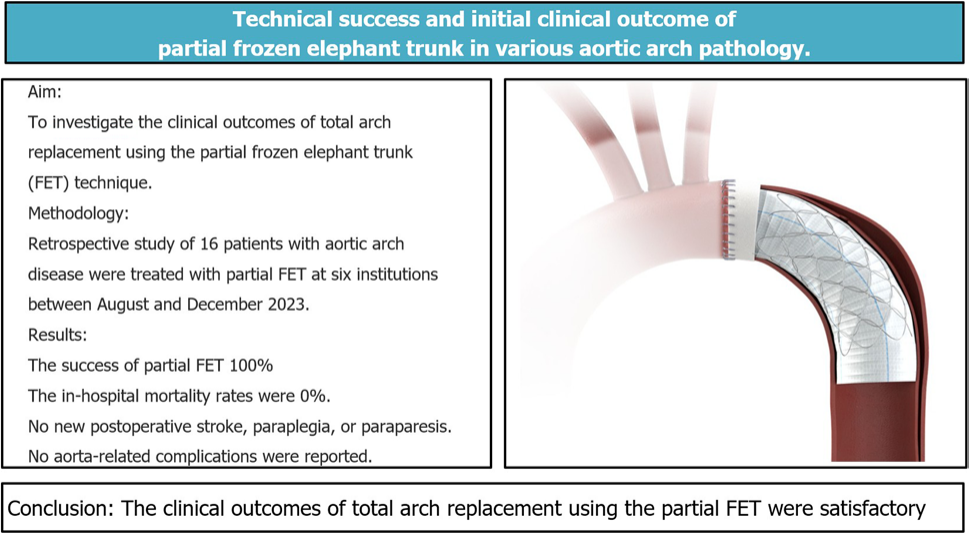

**Supplementary Information:**

The online version contains supplementary material available at 10.1007/s12055-025-02054-y.

## Introduction

The frozen elephant trunk (FET) technique is widely used to enhance aortic remodeling in patients with acute type A aortic dissection [1]. FET can repair the ascending aorta, aortic arch, and descending aorta at one stage, thereby reducing the risk of reintervention and improving downstream blood flow. However, FET is associated with complications such as postoperative spinal cord injury and stent graft–induced new entry (SINE). Recently, concerns regarding thrombus formation in FETs have gained attention. To address these issues, shorter FETs with reduced radial force have been developed, featuring the stent tip that is completely covered by the short graft. Here, we present the initial clinical outcomes of total arch replacement using this newly designed partial FET implant.

## Methods

### Ethics statement

The study was approved by the Medical Ethics Committee of Hamamatsu Rosai Hospital (approval number: 2538; date of approval: 25 October 2023) and conducted in accordance with the Declaration of Helsinki and the World Medical Association (WMA) Declaration of Taipei. As this was a non-invasive observational study in which measures were taken to ensure that subjects could not be identified, the requirement for informed consent was waived.

### Study design

This study is a multicenter, non-blinded, non-randomized assessment of the safety of open surgical repair of the aortic arch and descending aorta utilizing total arch replacement using a newly designed FET.

### Study population

Sixteen consecutive patients who underwent total arch replacement using the new FET technique for the treatment of aortic arch disease between August and December 2023 were examined. All the centers involved in this study were actively performing aortic emergency surgeries and had more than three surgeons with Japanese board certification of cardiovascular surgery. All patients provided written informed consent for the procedure. There were no exclusion criteria for this study.

### Endpoints

The primary endpoint of this study was to evaluate the safety and effectiveness of this partial FET in the treatment of aortic arch disease. The success of device implantation was the primary endpoint, and the secondary endpoints included mortality and the occurrence of aortic events, such as the development of a distal SINE. Follow-up is conducted every 6 months at the outpatient clinic employing computed tomography (CT) scan for assessment. Surgical variables examined cardiopulmonary bypass time, aortic cross-clamp time, and corporeal hypothermic circulatory arrest time. Morbidities are classified as follows: temporary neurological dysfunction was characterized by severe delirium, confusion, agitation, and memory disturbances, all of which were resolved prior to patient discharge. Permanent neurological dysfunction was defined as brain impairment at the time of discharge, with new postoperative lesions identified through CT or magnetic resonance imaging. Spinal cord ischemia presented as paraplegia and paraparesis, with or without accompanying bladder and rectal dysfunction. Cardiac complications were identified as perioperative myocardial infarction and low cardiac output necessitating intra-aortic balloon pumping or intravenous norepinephrine or epinephrine at doses exceeding 0.1 µg/kg/min. Respiratory failure was defined as the need for mechanical ventilation for more than 3 days, reintubation, or the requirement for tracheotomy. Acute renal failure was characterized as an increase in serum creatinine levels to 2.0 mg/dL in patients with preoperative serum creatinine levels below 1.0 mg/dL, elevation exceeding 100% of the preoperative level in patients with serum creatinine levels above 1.0 mg/dL, or the necessity for hemodialysis. Infection was defined as severe wound infection medias, including mediastinitis, graft infection, or sepsis.

### Data acquisition

Detailed patient information, encompassing age, sex, weight, types of surgical procedures, laboratory blood test results, perioperative blood transfusions, volumes of bleeding, and instances of re-exploration due to bleeding, were collected utilizing an electronic medical and anesthesia database system.

### Surgical procedures

The surgical approach to the aortic arch was performed via a median sternotomy, during which cardiopulmonary bypass was established with an arterial cannula placed in the ascending aorta or aortic arch, with venous drainage obtained from the right atrium or the superior and inferior vena cavae. Patients were cooled to a bladder or rectal temperature ranging from 25 to 28 °C. Following this cooling phase, hypothermic circulatory arrest and cardioplegic arrest were initiated. Once the transverse arch was opened, antegrade selective cerebral perfusion was commenced to ensure adequate brain perfusion. Arterial pressure was maintained within the range of 30–50 mmHg, with a flow rate of 10–12 mL/kg/min.

### Total arch replacement with the newly designed FET

**Fig. 1:**
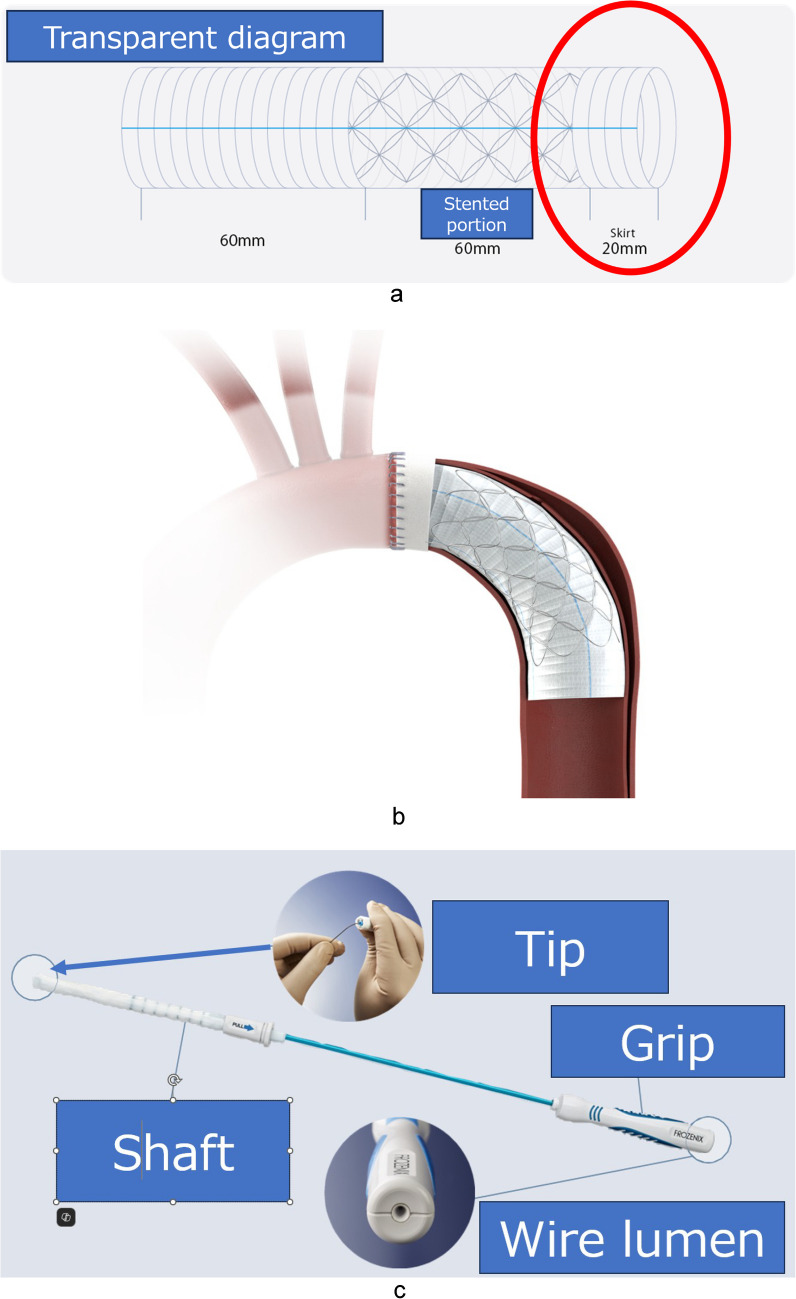
J Graft Frozenix Partial ET. **a** Transparent diagram of the Frozenix partial. It comprised a stented portion of 6 cm proximally and an unstented portion of 2 cm (encircled by a red line). **b** Schematic presentation of the implantation within the descending aorta in conjunction with total arch replacement utilizing four-branched Dacron graft. **c** The product features of the J Graft Frozenix Partial ET include a malleable shaft that allows surgeons to bend the device for insertion and attachment to the anatomy of the descending aorta. Distinct markers are present at 1-cm intervals to facilitate precise implantation. Additionally, a guide wire lumen is available for use in case of a tortuous aorta and/or a stenosed true lumen

The surgical procedure of total arch replacement with FET was conducted with the newly designed FET, J graft Frozenix Partial ET (Japan Lifeline, Tokyo, Japan) (Fig. 1). This approach aimed to facilitate the dilatation of the true lumen and the closure of the entry to the false lumen. The graft material is composed of polyester, and the stent material is nickel-titanium alloy. Distal aortic anastomoses were executed in Zone 3. The FET employed in this study features a 6-cm stented segment and a 2-cm distal non-stented section made solely of Dacron fabric portion with diameters ranging from 23 to 31 mm. This design is intended to minimize the spring back and radial forces by 43% and 29%, respectively, thereby reducing the SINE and facilitating the anastomosis between the unstented portion and the new graft second during the subsequent surgery for descending and/or thoracoabdominal aortic disease via a left thoracotomy. The sizing of the FET was based on specific criteria: approximately 90% of whole aorta in cases of acute dissection, approximately 100% of the true lumen calculated by dividing its circumference by 3.14 in chronic dissection, and 110% of the preoperative diameter in cases of aneurysm. The proximal end of the FET was secured to the surgically trimmed anastomotic site of Zone 3, and four-branched Dacron graft was anastomosed for total arch replacement and partial FET integration. The procedural details for the insertion and implantation of the partial FET are as follows: (1) bending the distal portion of the FET to conform the curvature of the aortic arch and the descending aorta; (2) inserting the FET into the descending aorta, if necessary, using a retrograde guidewire, or with the visual assistance of transesophageal echocardiography; (3) making fine positional adjustment to the proximal end of the FET, ensuring it is slightly distal to the trimmed anastomotic site; and (4) gently retracting the soft sheath to deploy the FET at the target location. Upon the completion of distal anastomosis involving the four-branched Dacron graft and partial FET, cardiopulmonary bypass with rewarming was initiated. Subsequently, the subclavian artery was anastomosed to a branch of the Dacron graft. Concurrent procedures, including root reconstruction and coronary artery bypass grafting, were performed at this stage. Following the completion of the proximal anastomosis, the aortic cross-clamp was released, and the reconstruction of the left carotid and brachiocephalic artery was carried out in this order.

### Data collection

Patient data were collected and subsequently transferred to Hamamatsu Rosai Hospital for comprehensive analysis.

### Statistical analysis

The statistical software JMP version 16.0.0 (SAS Institute Inc., Cary, NC, USA) was utilized for all statistical analyses. All the numerical data is presented as mean ± standard deviation.

## Results

**Fig. 2:**
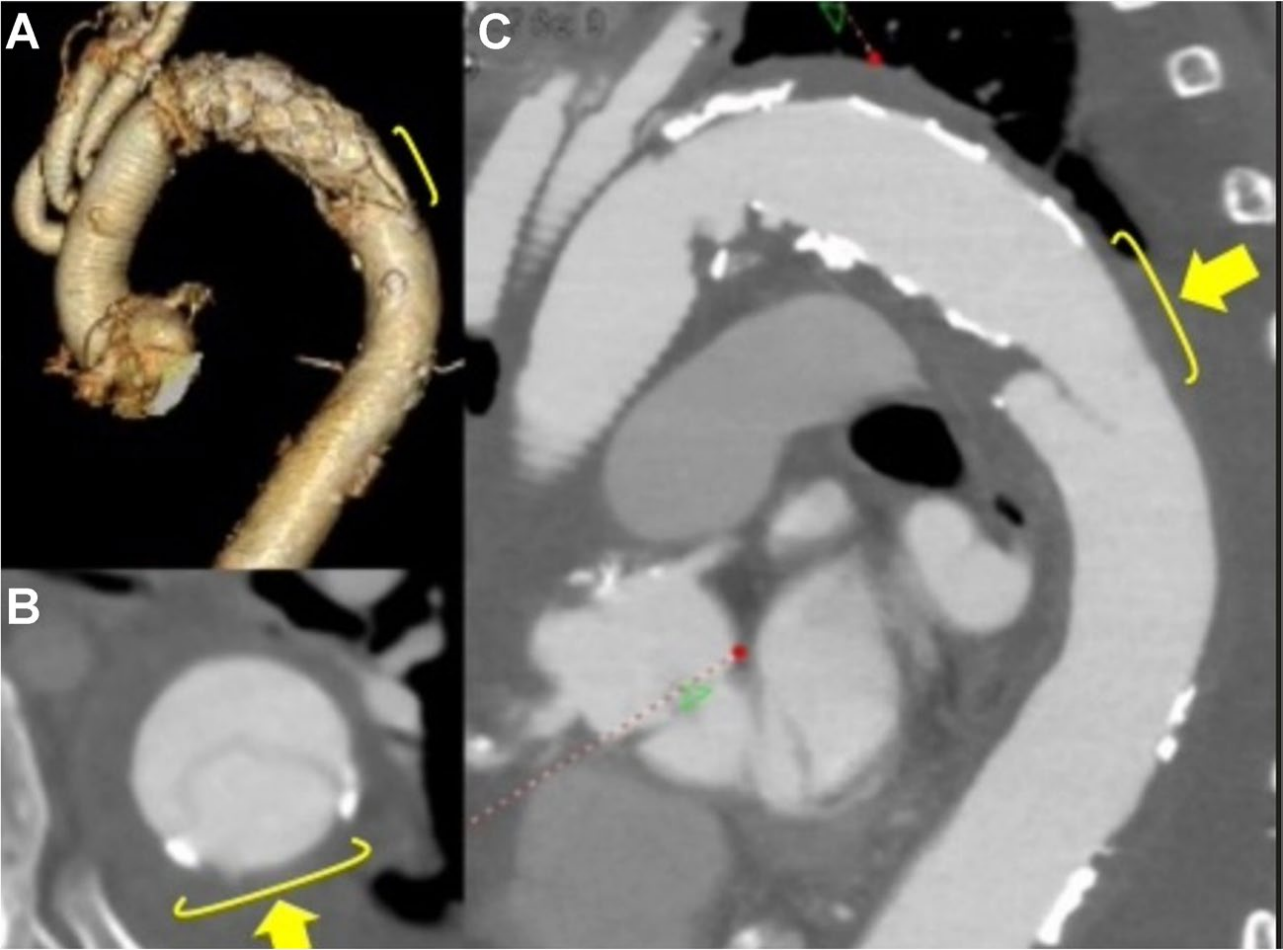
Representative presentation of postoperative aortic computed tomography angiogram featuring J Graft Frozenix Partial ET. **A** Three-dimensional reconstruction, **B** axial view, and **C** sagittal view. The yellow arrows and lines denote the distal unstented segment, which exhibits a slightly tapered configuration and demonstrates adequate contact with the major curvature side of the aorta

**Fig. 3:**
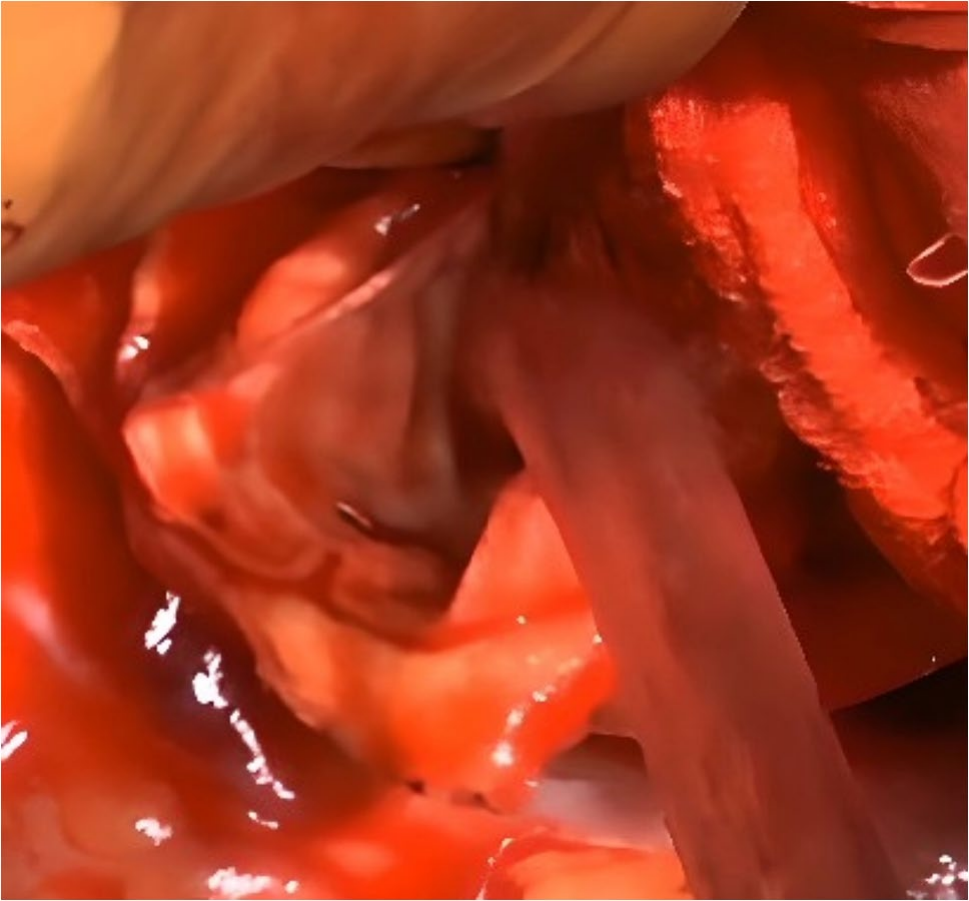
Representative image of the distal portion of the J Graft Frozenix Partial ET via left thoracotomy. The distal unstented segment is distinctly observable and appears to be suitable for subsequent anastomosis with a new graft via left thoracotomy during the procedure of thoracoabdominal aortic replacement

Background information and detailed characteristics of patients are summarized in Table 1. The mean age of the patients was 62 ± 10 years, with eight being female. The indications of surgical procedures included acute aortic dissection in ten patients, chronic aortic dissection in five patients, and anastomotic pseudoaneurysm in one patient. Among the 16 patients, two underwent reoperation. The size of the FET was 25.3 ± 1.9 mm. Preoperative malperfusion was noted in two patients, one exhibiting coronary symptoms and the other presenting cerebral symptoms. Total arch replacement utilizing partial FET was technically successful in all patients. Concomitant procedures included valve-sparing aortic root replacement in two patients, the Bentall procedure in one patient, and coronary artery bypass grafting (CABG) in one patient. The circulatory arrest time, aortic cross-clamp time, and cardiopulmonary bypass time were 62 ± 17, 176 ± 66, and 271 ± 65 min, respectively. Postoperative pressure gradient between the upper and lower extremities was observed in one patient with chronic aortic dissection, where the true lumen was severely stenosed, and the pressure gradient was not attributed to an unstented portion of the FET. This pressure gradient was resolved within weeks. The 30-day and in-hospital mortality rates were 0%. There are no new cases of postoperative stroke, paraplegia, or paraparesis. There were no aorta-related complications, such as distal SINE and intraluminal thromboembolism, in the partial FET (Fig. 2). With a follow-up rate of 100% and the follow-up period of 25.2 ± 0.8 months, no mortality and morbidity, including SINE, were observed. No graft thrombosis nor roll-back phenomena of the unstented portion of the partial FET were noted. No patient needed the secondary thoracic stent graft implantation. One patient with coexisting descending and thoracoabdominal aortic dilatation underwent thoracoabdominal aortic replacement 3 months after the initial surgery. During the second operation, an excellent surgical view of the distal unstented portion of the partial FET was obtained through left thoracotomy (Fig. 3 and Supplemental Video File).

**Table 1:** Background information and detailed characteristics of patients undergoing partial FET implantation

Number of the patients	16
Preoperative diagnosis	Acute aortic dissection 10 Chronic aortic dissection 5 Anastomotic pseudoaneurysm 1
Size of the partial FET used	25.2 ± 1.8 mm (23 mm: 4, 25 mm: 7, 27 mm: 4, 29 mm: 1)
Diameter of maximum long axis at the distal portion of the stent	25.4 ± 3.4 mm
Diameter of minimum short axis at the distal portion of the stent	20.1 ± 3.7 mm
Postoperative status of the false lumen	Thrombosed: 15

## Discussion

The original Frozenix model (Japan Lifeline, Tokyo, Japan) was introduced to the Japanese market in 2014. Since its introduction, it has been used in over 30,000 cases without significant complications [2]. The Frozenix partial examined in this study represents an additional iteration within the Frozenix series. As previously noted, the Frozenix partial employed in this study possesses several distinctive features.

First, shortness (6 cm stented length). In the initial clinical series of conventional Frozenix, approximately 24% of cases involved a stented length of 6 cm when utilized in aortic dissection [3]. Shorter stented lengths may mitigate the risk of spinal cord injury. The length of the FET significantly influences spinal cord damage; conversely, an increase in FET length correlates with enhanced true lumen expansion and a higher rate of false lumen occlusion. We posit that prioritizing the reduction of spinal cord injury risk is paramount, as false lumen occlusion can be addressed through additional stent graft implantation in the postoperative period. Should the false lumen remain unoccluded and expand during the chronic phase, it may necessitate intervention via open surgery through left thoracotomy. Recently, the phenomenon of thrombus formation within the FET has emerged as a concern [4–8]. The etiology of this thrombus formation remains unclear; however, factors such as the morphology of the FET and the stagnation of flow at its periphery have been pointed out as potential contributors. Although Frozenix is characterized as an endoskeletal FET with multiple circular metallic stents made from nickel-titanium, its distinctive stent morphology facilitates uniform and continuous expansion, thereby preventing the formation of a wavy internal structure. Notably, there have been no reported instances of intraluminal thrombosis within the FET across more than 30,000 cases of the Frozenix series, suggesting that the likelihood of thrombus formation within this FET is relatively low [1].

Second, the design incorporates a 2-cm stentless graft segment at the peripheral end, which may mitigate the impact of the stent on the aortic intima and decrease the likelihood of SINE. The reported incidence of distal SINE is 12.7%, with a range of 9.1–60% [9] while the incidence associated with the conventional Frozenix device is reported to be 14.1%, comparable to other types of FET [10]. Nevertheless, implementing preventive measures against this complication remains a critical concern. This design has already been documented in conjunction with FETs from various manufacturers and has been shown to reduce the incidence of SINEs [11, 12]. The differing lengths of previously reported FETs with similar modifications, as well as the more caudal positioning of the end of the inserted FET, may contribute to a decreased incidence of SINEs. Okamura et al. have shared their experiences utilizing a handmade modification of the Frozenix device based on this principle [13]. Furthermore, this stentless segment serves as the anastomosis site for graft-graft anastomosis in cases where aneurysmal changes progress in the descending and thoracoabdominal aorta [14]. In such a scenario, surgery is conducted via left thoracotomy. With the newly designed FET, graft-graft anastomosis was successfully completed without interference from the metallic stent. The formation of pseudoaneurysm has been reported between the FET and the new vascular prosthesis, in which polypropylene sutures engage the metallic structure of the FET [15]. Although pseudoaneurysm complications may occur due to other factors such as suture loosening or material deterioration [16, 17], this device theoretically alleviates concerns regarding these potential complications in the future.

Third, the elasticity of the stent has been diminished in comparison to that of the original Frozenix model. In the context of acute aortic dissection, the stent is engineered to effectively mitigate the risk of damage to the delicate aortic walls. However, in chronic dissection, the potential for the stent graft to tear may arise, contingent upon the integrity of the dissected septum, even if the expansion force of the stent graft is reduced. Consequently, this product may not be an optimal choice for chronic dissection, particularly with the aim of decreasing the probability of SINE. Insufficient dilatation of the stent graft may lead to inadequate expansion of the true lumen.

One concern regarding the surgical procedure is that anastomosis between the distal unstented portion of the Frozenix partial and the new prosthesis via the subsequent left thoracotomy may not be assured in instances of aortic disease progression affecting the descending aorta. The distal unstented segment may collapse, become intorted, or twist, which could render a subsequent anastomosis impractical. To evaluate this worst-case scenario, we conducted an experiment using a 40-kg swine model and assessed the feasibility of anastomosing the distal segment. Our findings indicated that this segment, measuring as short as 2 cm, was indeed suitable for anastomosis.

A notable concern pertains to the weak radial force. The absence of exclusion criteria in this study raises questions, particularly in light of chronic aortic dissection that exhibited temporarily diminished blood pressure in the lower extremities, although this issue was resolved during the index hospitalization. Given that partial FET is a novel device with limited prior experience, it would have been prudent to exclude certain cases from this study, specifically, patients with severe comorbidities or critical illnesses, those presenting with acute dissection accompanied by significant distal malperfusion, or individuals with multiple reentry tears who may deserve longer FET devices for proper true lumen expansion. Additionally, patients with aneurysmal disease of the aortic arch and descending thoracic aorta could potentially benefit from single-stage longer FET devices, as could chronic dissection patients with severely stenosed true lumens, as exemplified by the aforementioned case. Nevertheless, this study employed partial FET across six centers without implementing any exclusion criteria, resulting in clinically favorable outcomes.

### Limitations

This study acknowledges several limitations. First, it is designed as a prospective, multicenter, non-blind, non-randomized investigation. Second, the sample size of patients enrolled in the study is limited due to the recent introduction of the partial FET to the market. Consequently, there exists a potential risk of selection bias and various confounding factors, including performance and assessment bias. Thirdly, the etiology of the aortic arch disease is heterogeneous. Despite these limitations, this study effectively demonstrated the safety and efficacy of this novel FET in the context of aortic arch disease. Lastly, it is noteworthy that no cases requiring secondary thoracic stent graft implantation were encountered during this study. Therefore, the feasibility of utilizing partial FET for secondary thoracic stent graft remains uncertain, even though the unstented portion of the partial FET measures only 2 cm.

## Conclusions

The clinical outcomes of total arch replacement utilizing the partial FET procedure have been deemed acceptable. Nevertheless, it is imperative for surgeons to remain vigilant regarding the inherent radial and spring back forces when employing this device in cases of chronic aortic dissection.

## Supplementary Information

Below is the link to the electronic supplementary material.

Supplementary file1 (JPG 241 KB)

Supplementary file2 (MP4 65685 KB)

## Data Availability

All relevant data are within the manuscript and its supporting information files.
