# Supplementary figures and images for: Technical success and initial clinical outcome of partial frozen elephant trunk in various aortic arch pathology
Source: Indian J Thorac Cardiovasc Surg. 2025 Sep 15;41(11):1560–7. doi: 10.1007/s12055-025-02054-y (PMC12549462; doi:10.1007/s12055-025-02054-y)

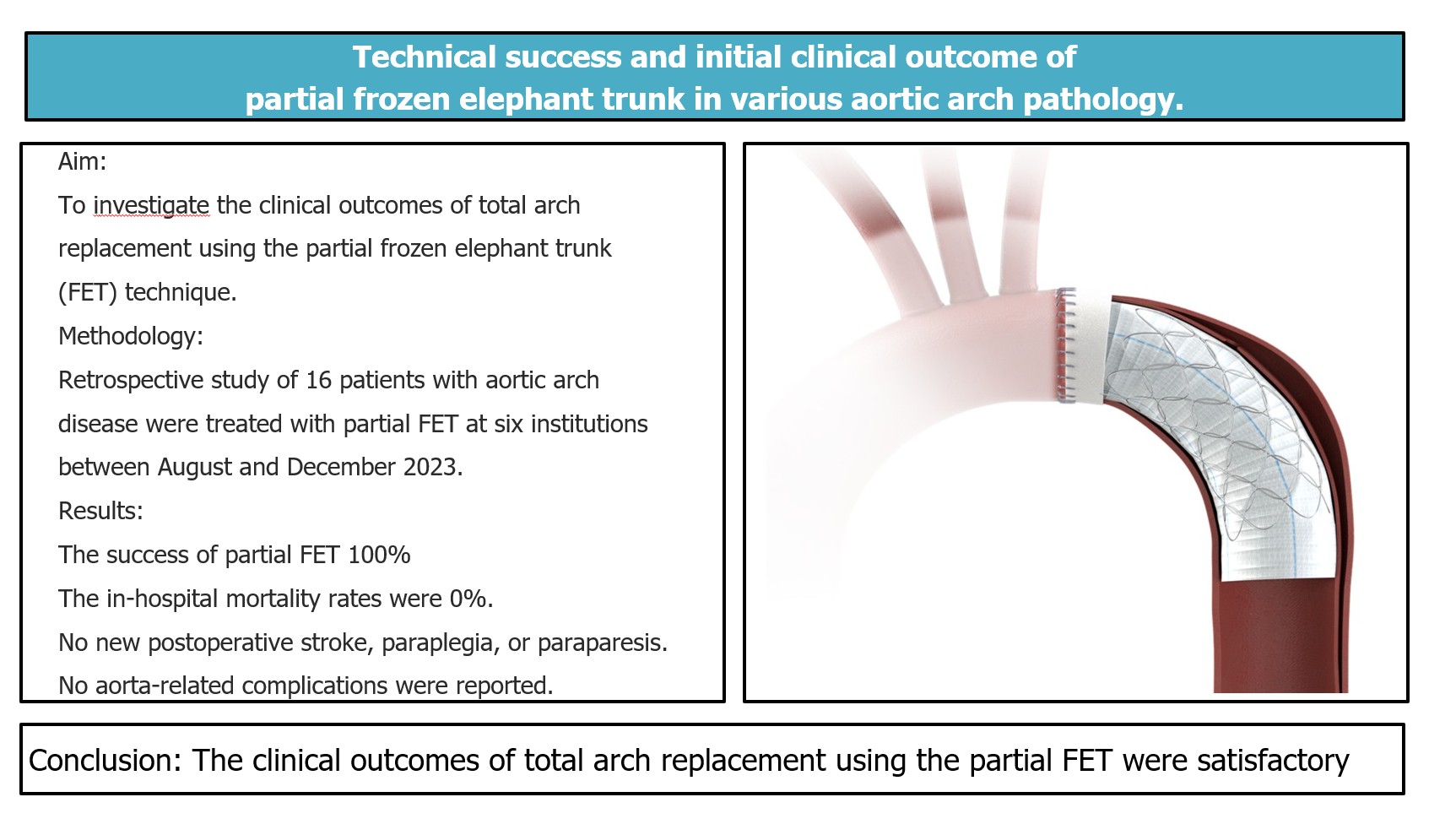

Supplement: Supplementary file 1 — Supplementary file1 (JPG 241 KB) [file 12055_2025_2054_MOESM1_ESM.jpg]
